# Complete Genome Sequence of *Mesorhizobium ciceri* bv. biserrulae Strain WSM1284, an Efficient Nitrogen-Fixing Microsymbiont of the Pasture Legume *Biserrula pelecinus*

**DOI:** 10.1128/genomeA.00514-16

**Published:** 2016-06-09

**Authors:** Timothy Haskett, Penghao Wang, Joshua Ramsay, Graham O’Hara, Wayne Reeve, John Howieson, Jason Terpolilli

**Affiliations:** a Centre for Rhizobium Studies, Murdoch University, Perth, Australia; b School of Biomedical Sciences and Curtin Health Innovation Research Institute, Curtin University, Perth, Australia

## Abstract

We report the complete genome sequence of *Mesorhizobium ciceri* bv. biserrulae strain WSM1284, a nitrogen-fixing microsymbiont of the pasture legume *Biserrula pelecinus*. The genome consists of 6.88 Mb distributed between a single chromosome (6.33 Mb) and a single plasmid (0.55 Mb).

## GENOME ANNOUNCEMENT

*Biserrula pelecinus*, an annual herbaceous legume native to the Mediterranean basin, was introduced into Australian agriculture in 1994 (1). As a pasture legume, *B. pelecinus* is well suited to Australian conditions because it grows well in acidic soils, is drought and insect tolerant, has abundant seed production, and is easy to harvest (1, 2). *B. pelecinus* forms a nitrogen-fixing symbiosis with soil bacteria within the genus *Mesorhizobium*. Australian soils were initially devoid of any *B. pelecinus*–nodulating organisms (1), leading to a search for effective N_2_-fixing inoculants. *Mesorhizobium ciceri* bv. biserrulae strain WSM1284 was isolated from a nodule of *B. pelecinus* growing at Siniscola, in Sardinia, Italy (3). Similar to other *B. pelecinus* isolates, such as *M. ciceri* bv. biserrulae WSM1271 (4) and WSM1497 (5), WSM1284 is an effective microsymbiont on its host of origin and does not nodulate *Cicer arietinum* (chickpea). However, unlike WSM1271 and WSM1497, WSM1284 has a broad host range, being capable of nodulating species of *Astragalus*, *Dorycnium*, *Glycyrrhiza*, *Leucaena*, *Lotus*, and *Ornithopus* (5–7). The complete genome sequence of this organism will therefore facilitate work to understand the molecular basis of this broad-host range.

WSM1284 genomic DNA was extracted from a tryptone-yeast-grown culture (8) using a phenol-chloroform method as previously described (9). Whole-genome sequencing was performed by Macrogen (South Korea), using both Pacific BioSciences (PacBio) single-molecule real-time sequencing and Illumina HiSeq 2500 technology. Post-filter, PacBio sequencing generated 1,210,355,345 bases consisting of 102,356 trimmed reads, with Illumina sequencing generating an additional 2,140,158,286 bases constituting 21,189,686 paired-end reads. Raw Illumina reads were analyzed using FastQC version 0.10.1 (http://www.bioinformatics.babraham.ac.uk/projects/fastqc) and adaptors were removed by comparison against a comprehensive in-house adaptor sequence library. PacBio subreads were assessed using in-house software, and reads were automatically error-corrected in the assembly process.

Filtered Illumina and PacBio reads were assembled *de novo* using the hybrid approach of SPAdes assembler version 3.6.2 (10), with the number of mismatches and short indels reduced by incurring SPAdes’s postprocessing module MismatchCorrector, utilizing the BWA tool (11). The assembly obtained was scaffolded using SSPACE version 3.0 (12) and annotated using the NCBI Prokaryotic Genome Annotation Pipeline (PGAP) (http://www.ncbi.nlm.nih.gov/genomes/static/Pipeline.html). The genome consists of 6,880,454 bp with an average GC content of 62.51%. There are 6,499 coding sequences distributed between a single circular chromosome of 6,326,813 bp and a single plasmid of 553,641 bp.

The majority of WSM1284 genes required for nodulation (*nod*) and nitrogen fixation (*nif* and *fix*) were identified within a 539-kb region of the chromosome. Within this region, the presence of genes encoding a putative type IV secretion system, conjugative relaxase, biotin, and nicotinate biosynthetic clusters and genes with homology to a quorum sensing system shown to regulate integrative and conjugative element excision and transfer in *Mesorhizobium loti* R7A (13–16) indicates that WSM1284 may harbor a symbiosis island. However, the absence of phage-like P4 integrases and direct-repeat attachment sites required for symbiosis island excision (17) suggests that this region is either nonmobile or utilizes a novel mechanism of excision prior to self-transmission. Whether the WSM1284 symbiosis island is capable of conjugal transfer is a question currently being investigated.

### Nucleotide sequence accession numbers.

The nucleotide sequence of the complete genome of WSM1284 has been deposited in GenBank under the accession numbers CP015064 (chromosome) and CP015065 (plasmid pMc1284).
